# Real‐Time in Vivo Monitoring of Circulating Endometrial Cells: Uncovering Systemic Dissemination and Temporal Fluctuations in Endometriosis

**DOI:** 10.1002/advs.76327

**Published:** 2026-06-26

**Authors:** Shang Wang, Chenyu Hou, Buyun Li, Hongyan Cheng, Xue Ye, Honglan Zhu, Yanmin Li, Rui Chen, Sihan Dong, Yi Li, Huiping Liu, Chen Zhang, Rui Zhang, Hongyi Hou, Xunbin Wei, Xiaohong Chang

**Affiliations:** ^1^ Department of Obstetrics and Gynecology Peking University People's Hospital Beijing China; ^2^ Beijing Key Laboratory of Female Oncofertility Beijing China; ^3^ Key Laboratory of Carcinogenesis and Translational Research (Ministry of Education/Beijing) Peking University Cancer Hospital & Institute Beijing China; ^4^ Biomedical Engineering Center Institute of Advanced Clinical Medicine Peking University Beijing China; ^5^ Peking University International Cancer Institute Beijing China; ^6^ Institute of Medical Technology and Cancer Hospital Peking University Beijing China; ^7^ Beijing Advanced Center of Cellular Homeostasis and Aging‐Related Diseases Beijing China

**Keywords:** circulating endometrial cells (CECs), endometriosis, in vivo cellular dissemination, in vivo dynamic tracing, pseudo‐menstrual mouse models

## Abstract

Endometriosis is a common gynecological disorder characterized by the ectopic growth of endometrium‐like tissue outside the uterine cavity and is associated with pelvic pain, infertility, and reduced quality of life. Although several theories have been proposed, the mechanisms by which endometrial cells disseminate and establish ectopic lesions remain incompletely understood. Here, we developed a dual‐color pseudo‐menstrual mouse model that enables real‐time in vivo tracking of circulating endometrial cells (CECs) while distinguishing their tissue origin as uterine‐derived or lesion‐derived. Using in vivo flow cytometry and two‐photon intravital microscopy, we found that both uterine‐derived and lesion‐derived CECs can enter the circulation and are detected at higher levels during menstruation‐like breakdown, potentially in association with local vascular remodeling as suggested by intravital imaging observations. These cells could localize to distant organs, including the lungs and other extrapelvic organs. In addition, both CEC populations exhibited three distinct daily peak periods, and CEC dynamics were significantly associated with fluctuations in serum prolactin levels. In conclusion, these findings provide in vivo support for hematogenous dissemination in endometriosis and suggest that hormonal rhythmicity may regulate CEC dynamics, with potential diagnostic and therapeutic implications.

## Graphical abstract

A dual‐color pseudo‐menstrual mouse model enables real‐time tracing of circulating endometrial cells (CECs) from uterine and ectopic sources. The study reveals menstrual‐phase‐dependent, burst‐like CEC release, distinct postoperative behaviors of different CEC origins, and a potential association with prolactin fluctuations, providing an in vivo framework for understanding CEC dissemination in endometriosis.

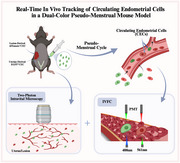

## 1 Introduction

Endometriosis (EM) is a chronic, estrogen‐dependent inflammatory disorder that affects approximately 10% of women of reproductive age [1]. Histologically, it is defined by the presence of endometrium‐like glands and stroma outside the uterine cavity, most commonly involving the ovaries and peritoneum; in more severe cases, distant organs such as the lungs, diaphragm, and central nervous system may also be affected [2, 3, 4, 5, 6]. Clinically, EM is frequently associated with pelvic pain, dysmenorrhea, dyspareunia, infertility, and fatigue, substantially impairing quality of life [7, 8]. Although the disease was first described more than a century ago, its pathogenesis remains incompletely understood.

The most widely accepted theory is Sampson's retrograde menstruation theory, proposed in the 1920s, which suggests that endometrial fragments reflux through the fallopian tubes into the pelvic cavity during menstruation and subsequently implant at ectopic sites [9]. However, retrograde menstruation is observed in up to 90% of menstruating women, whereas only a subset ultimately develops EM [10, 11]. In addition, this theory does not fully account for the formation of extrapelvic or even distant lesions, suggesting that mechanisms other than retrograde implantation may also contribute to disease dissemination [12, 13]. Among these, hematogenous dissemination has emerged as a mechanism of particular interest.

In recent years, circulating endometrial cells (CECs) have been detected in the peripheral blood of patients with EM [14], suggesting that endometrial‐related cells are able to enter the systemic circulation and providing new clues for investigating the role of blood‐borne cell migration in disease spread. Previous studies from our group and others have used microfluidic platforms and subtraction enrichment‐immunostaining fluorescence in situ hybridization (SE‐iFISH) to isolate and characterize CECs from patient blood samples [14, 15]. These analyses showed that CECs are positive for cytokeratin and CD10, display chromosomal aneuploidy, and lack hematopoietic markers, supporting their endometrial phenotype. Based on these findings, we propose the CEC hypothesis, namely that endometrial cells can enter the bloodstream and circulate as CECs, where they may act in concert with retrograde menstruation to contribute to the dissemination and progression of EM (Figure S1).

However, current studies of CECs rely largely on single‐time‐point analyses of peripheral blood, making it difficult to capture the temporal dynamics of CEC release across the menstrual cycle or circadian rhythm. More importantly, real‐time in vivo tracking of the entry, migration, and distribution of endometrial‐related cells is not feasible in humans. Progress in this field has also been limited by the lack of an appropriate animal model that can recapitulate menstruation while allowing real‐time monitoring of CEC behavior. Except for the spiny mouse, rodents undergo estrous rather than menstrual cycles and do not menstruate spontaneously, posing an inherent biological limitation [16, 17]. Although non‐human primates more closely resemble humans physiologically, their use is constrained by cost, availability, and ethical considerations [18, 19].

To address these challenges, we established a dual‐color pseudo‐menstrual mouse model. By combining hormonal priming with endometrial manipulation to mimic cyclical endometrial breakdown and bleeding, and by incorporating eutopic and ectopic endometrial tissues derived from EGFP and tdTomato transgenic mice, respectively, we were able to distinguish CECs of different origins and track them in real time. By further integrating in vivo flow cytometry (IVFC) with two‐photon intravital microscopy, we continuously quantified circulating fluorescent cells and visualized key events at both the vascular and tissue levels [20, 21, 22].

Using this platform, the present study aims to define the dynamic process by which endometrial cells enter the bloodstream, migrate, and distribute in vivo, to compare the contributions of eutopic and ectopic sources to the CEC population, and to further explore the release patterns of CECs and their potential hormonal regulation. Through these investigations, we seek to provide an experimental basis for evaluating the CEC hypothesis from an in vivo dynamic perspective and for clarifying the potential role of hematogenous dissemination in EM.

## 2 Results

### 2.1 Construction of the Dual‐Color Pseudo‐Menstrual Mouse Model

**FIGURE 1:**
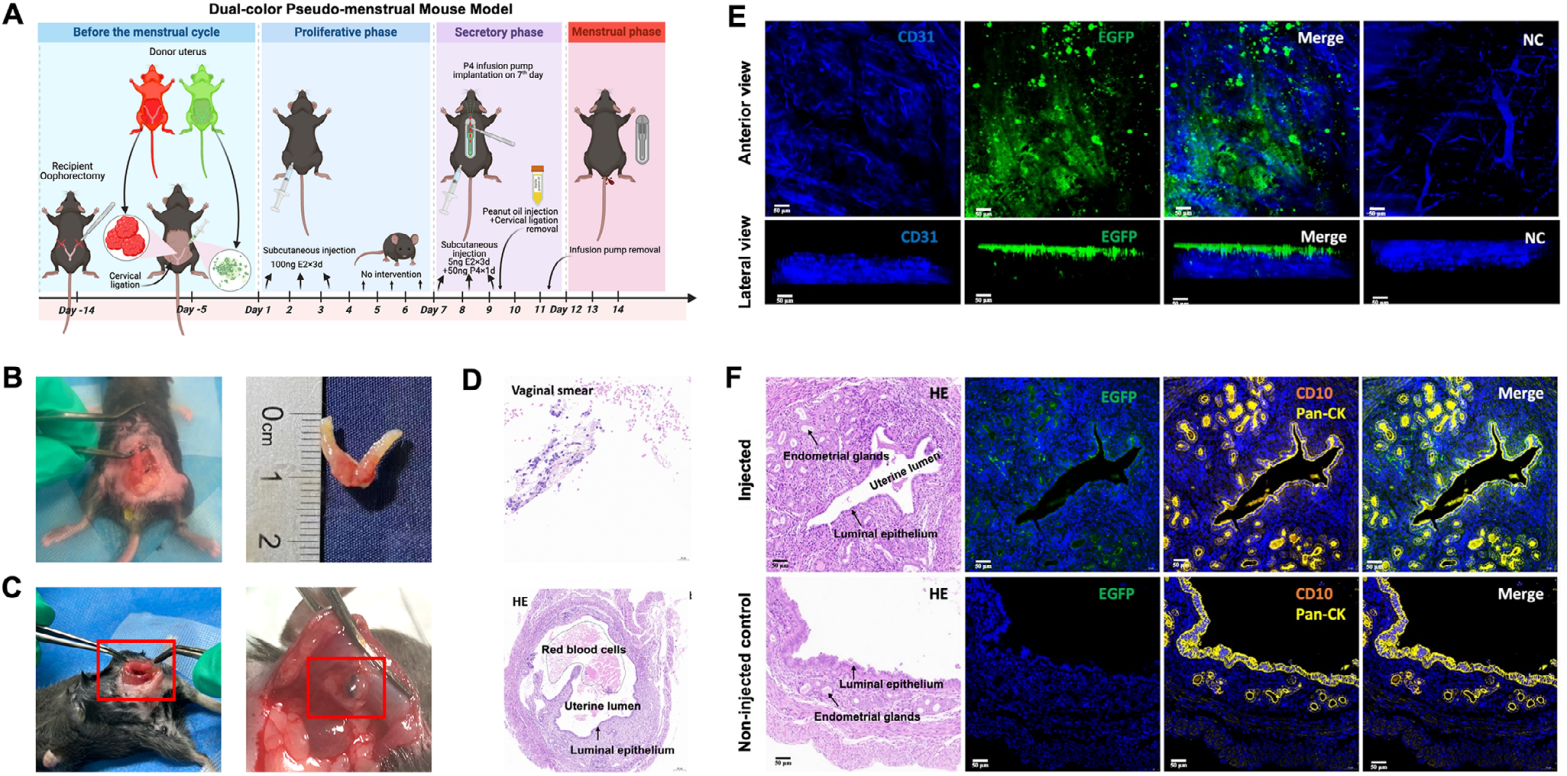
Construction and validation of the dual‐color pseudo‐menstrual mouse model. (A) Schematic overview of the construction of the dual‐color pseudo‐menstrual mouse model. (B) Gross appearance of the uterus during the menstrual phase, showing uterine enlargement, congestion, and bleeding. (C) Representative ectopic lesion at the abdominal wall suture site, where tdTomato‐derived endometrial tissue formed a blister‐like lesion. (D) H&E staining and vaginal smear analysis validating menstrual shedding in the model. (E) Two‐photon intravital microscopy of the uterine cavity (anterior and lateral views), showing EGFP^+^ cells distributed along the uterine surface. (F) Corresponding H&E staining and immunofluorescence images (EGFP, Pan‐CK, CD10, and DAPI) showing integration of EGFP^+^ cells into the endometrial structure.

To establish a robust in vivo model for studying endometriosis, we first developed an approach for transplanting endometrial tissues into recipient mice. Briefly, tdTomato^+^ endometrial tissue from transgenic donor mice was transplanted onto the abdominal wall of recipient mice, whereas EGFP^+^ endometrial tissue from another transgenic donor was enzymatically dissociated and injected into the uterine cavity of the same recipients, thereby generating a dual‐color mouse model. The mice were then subjected to sequential estrogen and progesterone treatment and hormone withdrawal following previously described pseudo‐menstrual protocols [23]. The overall workflow of model construction is outlined in Figure 1A, highlighting the key steps used to recapitulate key features of the disease.

Approximately 2 weeks after surgery, the uterus appeared markedly enlarged and hyperemic upon opening the abdominal cavity, providing the first gross evidence of successful model establishment (Figure 1B). In addition, a distinct blister‐like lesion was observed at the abdominal wall suture site, with a morphology consistent with endometriotic lesions (Figure 1C).

To further validate menstrual shedding in the model, we examined bleeding, a key hallmark of endometrial shedding. Both vaginal smears and uterine tissue sections showed abundant red blood cells, indicating that the model successfully recapitulated menstrual shedding (Figure 1D).

Finally, to enable real‐time visualization of cellular behavior, we performed two‐photon intravital microscopy to monitor EGFP^+^ cells within the uterine cavity. These cells formed a continuous layer along the uterine surface and showed integration with the underlying tissue, indicating incorporation into the endometrial structure (Figure 1E). This observation was further supported by multicolor immunofluorescence, which demonstrated a high density of EGFP^+^ cells within the uterine tissue (Figure 1F).

These results indicate that we successfully established a dual‐color pseudo‐menstrual mouse model. This model recapitulates key features of endometriosis and menstrual changes and provides an experimental platform for subsequent in vivo tracking of cells from distinct origins.

### 2.2 EGFP^+^ Endometrial Cells Integrated Into the Recipient Uterus and Were Shed During the Menstrual Phase

**FIGURE 2:**
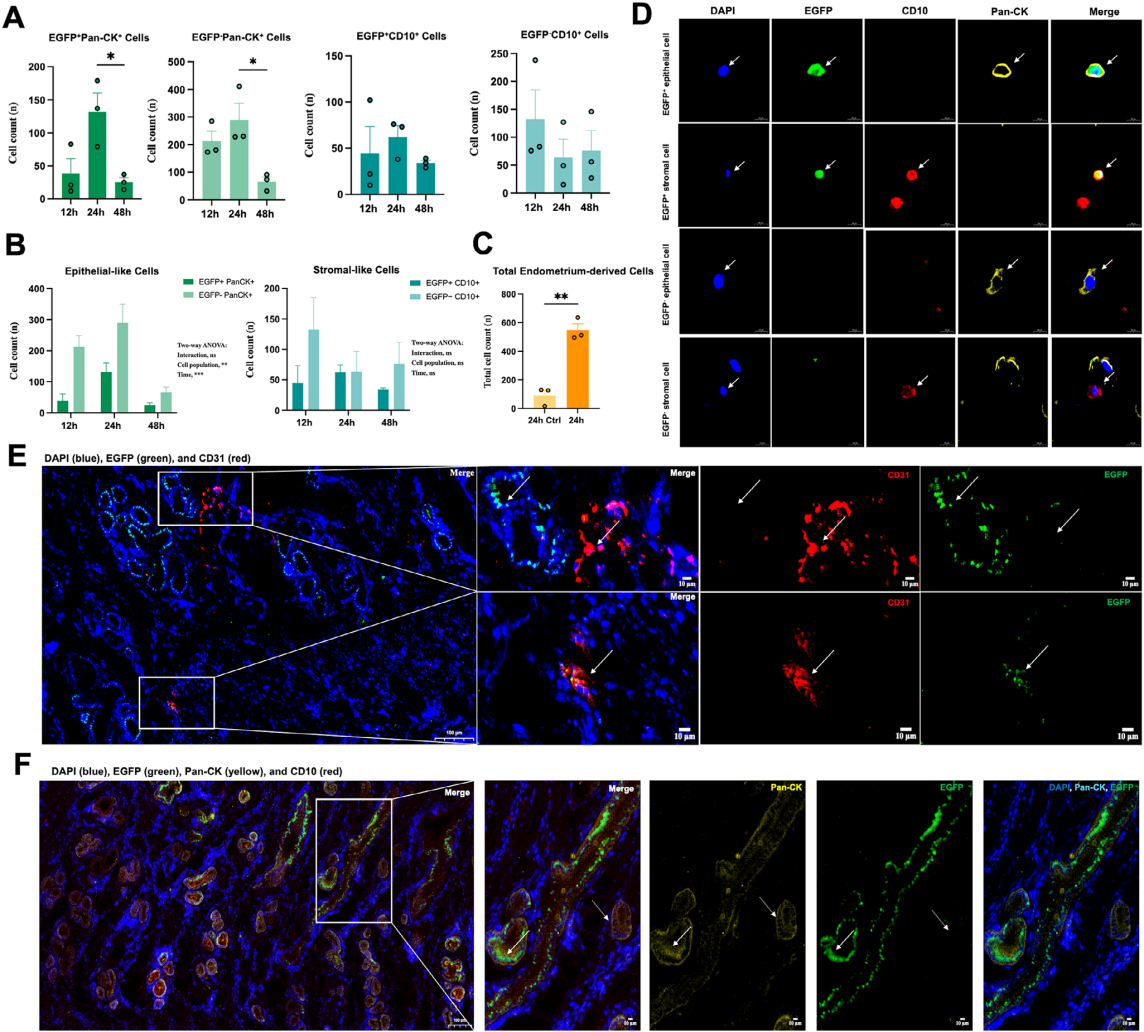
Menstrual‐like shedding and uterine integration of EGFP^+^ endometrial cells. (A) Quantification of EGFP^+^Pan‐CK^+^, EGFP^−^Pan‐CK^+^, EGFP^+^CD10^+^, and EGFP^−^CD10^+^ cells in uterine lavage samples at 12, 24, and 48 h after progesterone withdrawal. EGFP^+^Pan‐CK^+^ and EGFP^−^Pan‐CK^+^ cells were significantly higher at 24 h than at 48 h (*p* = 0.0296 and 0.0216, respectively), whereas EGFP^+^CD10^+^ and EGFP^−^CD10^+^ cells showed no significant differences (all *p* > 0.05). (B) Two‐way ANOVA analysis of EGFP^+^ and EGFP^−^ epithelial‐like and stromal‐like cells. In epithelial‐like cells, cell population and time were significant (*p* = 0.0013 and 0.0007), whereas the interaction was not (*p* = 0.1323). No significant effects were detected in stromal‐like cells (all *p* > 0.05). (C) Total endometrium‐derived cells in uterine lavage samples were significantly increased in the 24 h experimental group compared with the 24 h control group (*p* = 0.0016). (D) Representative multiplex immunofluorescence images of uterine lavage smear cells showing EGFP^+^ and EGFP^−^ epithelial‐like and stromal‐like cells. (E, F) Representative uterine sections showing localization of EGFP^+^ cells around or within CD31^+^ vascular structures and their integration into the endometrium, including Pan‐CK^+^ epithelial regions. Target cells were counted from 15 randomly selected nonoverlapping fields per uterine lavage smear, and the total count was used as one value per mouse. Data are presented as mean ± SEM (*n* = 3 per group/time point). 12, 24, and 48 h indicate experimental groups after progesterone withdrawal; 24 h Ctrl indicates the non‐withdrawal control group. *p < 0.05; **p < 0.01; ns, not significant.

To determine whether EGFP^+^ CECs in the pseudo‐menstrual model reflect authentic menstrual‐like shedding rather than bleeding alone, uterine lavage was collected at 12, 24, and 48 h after progesterone pump removal, with non‐withdrawal mice as controls. EGFP^+^ cells were readily detected among shed cells, together with Pan‐CK^+^ epithelial‐like and CD10^+^ stromal‐like cells. EGFP^−^Pan‐CK^+^ and EGFP^−^CD10^+^ cells were also observed, indicating that the lavage contained both exogenously introduced and endogenous endometrial cell populations (Figure 2D).

Quantitative analysis showed that both EGFP^+^Pan‐CK^+^ and EGFP^−^Pan‐CK^+^ cells changed dynamically after progesterone withdrawal, reached relatively higher levels at 24 h, and declined by 48 h. In both populations, the 24 h group was significantly higher than the 48 h group, whereas the remaining comparisons were not significant (p > 0.05). By contrast, neither EGFP^+^CD10^+^ nor EGFP^−^CD10^+^ cells differed significantly across time points (Figure 2A).

Two‐way ANOVA further showed that, in epithelial‐like cells, both cell population and time were significant factors, whereas their interaction was not, indicating similar temporal trends despite differences in absolute cell number. In stromal‐like cells, neither cell population, time, nor interaction was significant (Figure 2B). Consistently, the total number of endometrium‐derived cells was significantly higher in the 24 h experimental group than in the 24 h control group (*p* < 0.01) (Figure 2C).

Multiplex immunofluorescence of uterine sections showed that EGFP^+^ cells were located both around or within vascular structures and at sites distant from vessels, where they appeared integrated into the endometrium; notably, many localized to Pan‐CK^+^ epithelial regions (Figure 2E,F).

These findings support that exogenously introduced EGFP^+^ endometrial cells integrate into the endometrium and participate in authentic menstrual‐like shedding after progesterone withdrawal.

### 2.3 Real‐Time Tracking Revealed Menstrual‐Phase‐Specific Release of CECs

**FIGURE 3:**
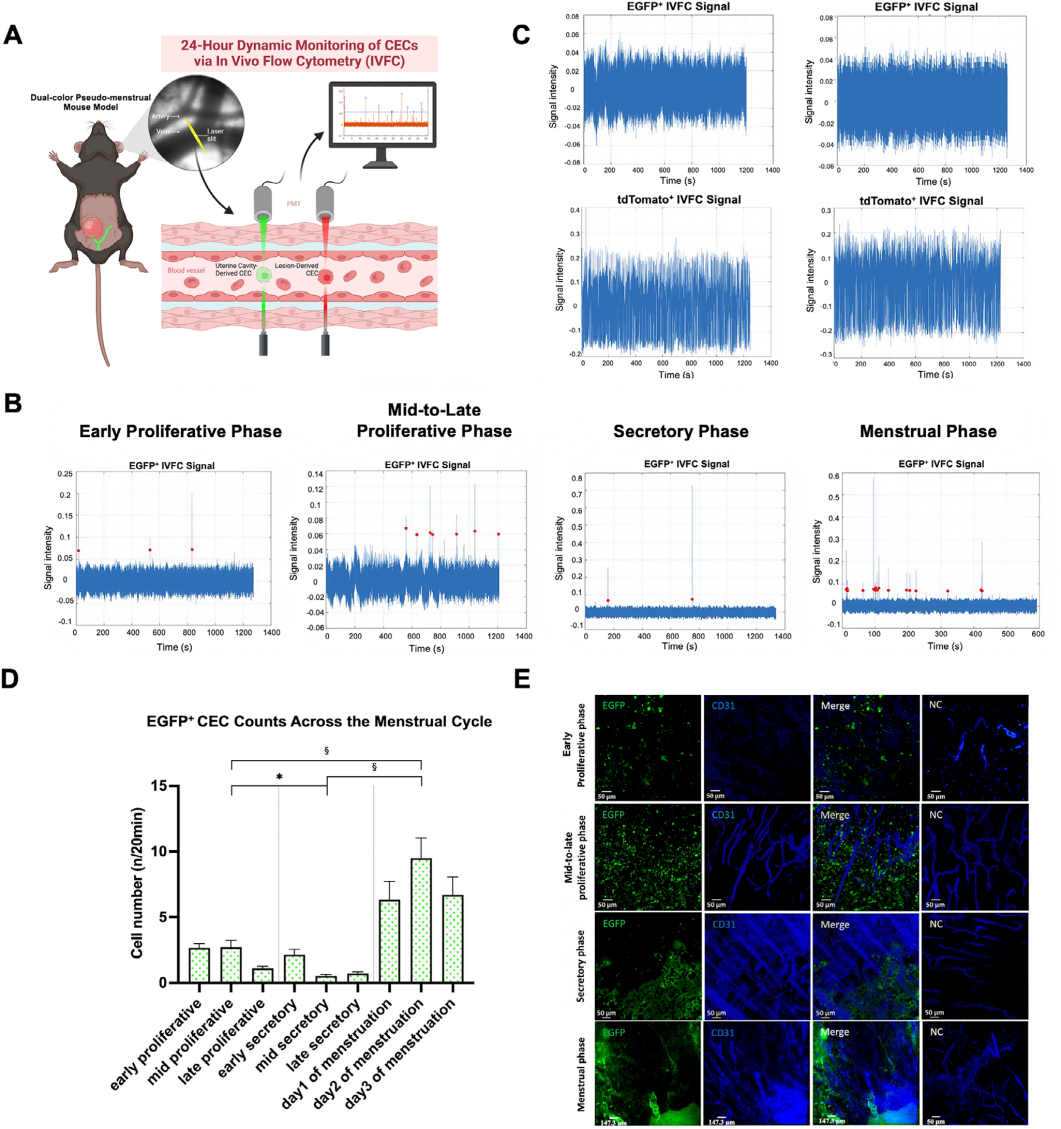
Real‐time monitoring of uterine‐derived EGFP^+^ CECs. (A) Schematic illustration of the in vivo flow cytometry (IVFC) system used for real‐time monitoring of CECs in the circulation. (B) Continuous IVFC monitoring of uterine‐derived EGFP^+^ CECs from day 1 to day 14, showing distinct cycle phases, including the early proliferative, mid‐to‐late proliferative, secretory, and menstrual phases. (C) No EGFP^+^ or tdTomato^+^ CEC signals were detected in the sham‐operated group or before the onset of the menstrual phase, supporting the specificity of the observed signals. (D) Quantification of uterine‐derived EGFP^+^ CECs across different cycle phases, showing significantly higher CEC counts during the menstrual phase than during the proliferative and secretory phases. Data are presented as mean ± SEM (*n* = 6 mice/phase; mixed‐effects model). (E) Two‐photon intravital microscopy showing endometrial vascular remodeling across different cycle phases and the close association of EGFP^+^ CECs with the vascular lumen. Blood vessels increased in number and diameter during the early and mid‐to‐late proliferative phases, reached maximal enlargement during the secretory phase, and showed focal disruption during the menstrual phase, accompanied by bleeding. A lower‐magnification view was used in the menstrual‐phase panels to better visualize the overall tissue disruption, whereas higher‐magnification images were used to show local EGFP^+^ and CD31^+^ fluorescence signals. NC, negative control. * *p* < 0.05, ^§^ *p* < 0.0001.

To investigate when and how endometrial cells enter the bloodstream under physiological conditions, we performed real‐time monitoring of circulating fluorescent cells in the pseudo‐menstrual mouse model using in vivo flow cytometry (IVFC) (Figure 3A).

First, to assess the sensitivity of IVFC for detecting endometrial cells in vivo, different numbers of hEM15A cells were injected into untreated C57BL/6 mice via the tail vein. The results showed that identifiable IVFC signals could still be detected even when the number of circulating endometrial cells was extremely low, at only single‐digit levels (Figures S2 and S3).

EGFP^+^ CECs were dynamically monitored across four phases of the cycle: the early proliferative phase, the mid‐to‐late proliferative phase, the secretory phase, and the menstrual phase (Figure 3B). As a negative‐control validation for IVFC detection, both EGFP and tdTomato channels were examined in sham‐operated mice and before the onset of menstrual shedding. Within the same 14‐day monitoring window, no fluorescent signals were detected in the sham‐operated control group. Likewise, no fluorescent signals were observed in any group before the onset of menstrual shedding (day −14 to day 0), supporting the temporal specificity and biological origin of the circulating cells detected (Figure 3C).

A marked increase in circulating EGFP^+^ CECs was observed during the menstrual phase, with an average of 7.51 cells/20 min. This level was significantly higher than that in the other phases, including 2.16 cells/20 min in the proliferative phase, and 1.60 cells/20 min in the secretory phase (*p* < 0.0001) (Figure 3D).

To investigate how CECs enter the bloodstream, we further performed two‐photon intravital microscopy of the endometrium during the menstrual phase. Endometrial vessels underwent marked dynamic remodeling during the pseudo‐menstrual cycle, characterized by vascular dilation and increased density in the secretory phase, followed by structural disorganization and focal loss of continuity during the menstrual phase (Figure 3E). We further observed EGFP^+^ CECs within the vascular lumen moving slowly with the bloodstream (Movie S1), supporting their presence in the circulation. These observations suggest that CEC entry into the bloodstream may be associated with endometrial vascular remodeling and focal barrier disruption during the menstrual phase.

To exclude the possibility that nonspecific cell entry into the bloodstream resulted from the modeling procedure or injection‐related injury, we performed a control experiment in which EGFP^+^ mouse‐derived fibroblasts were used in place of endometrial cells. In the fibroblast group, only occasional, extremely low‐frequency, scattered EGFP^+^ events were observed, with no positive signals detected during most monitoring periods and no stable or reproducible burst‐like release pattern identified (Figure S4A). These results suggest that the modeling procedure itself does not induce generalized entry of arbitrary exogenous cells into the peripheral circulation.

These findings suggest that EGFP^+^ CECs are preferentially detected in the bloodstream during the menstrual phase, possibly in association with hormone‐withdrawal‐induced endometrial breakdown. This menstrual‐phase‐dependent release pattern may provide a potential mechanistic explanation for the long‐range dissemination of endometrial cells in endometriosis.

### 2.4 Lesion‐Derived CECs Showed Similar Release Patterns but Lower Pulmonary Retention Than Uterine‐Derived CECs

**FIGURE 4:**
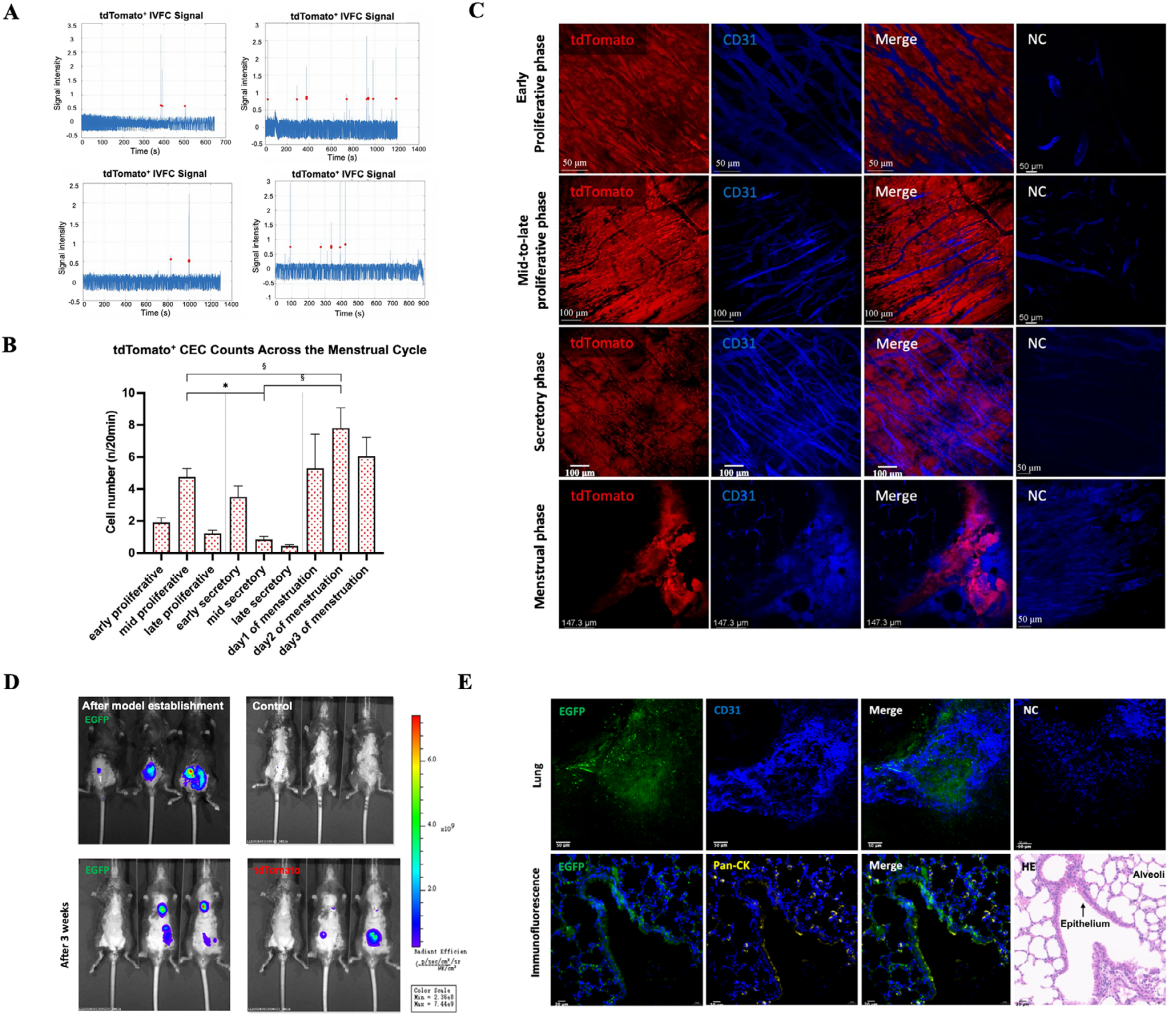
Lesion‐derived tdTomato^+^ CECs exhibit menstrual‐phase release, but lower pulmonary retention than endometrial‐origin CECs. (A) IVFC monitoring of tdTomato^+^ CECs from day 1 to day 14, showing CEC release during the early proliferative, mid‐to‐late proliferative, secretory, and menstrual phases. (B) Quantification of tdTomato^+^ cells in the bloodstream, showing a significant increase during the menstrual phase. Data are presented as mean ± SEM (*n* = 6 mice/phase; mixed‐effects model). (C) Two‐photon intravital microscopy of ectopic lesions showing phase‐dependent vascular remodeling and focal vascular discontinuity during the menstrual phase. (D) IVIS fluorescence imaging confirming systemic dissemination of EGFP^+^ cells, with significant accumulation in the lungs. (E) Histological analysis showing the presence of numerous EGFP^+^ cells in the lungs. Pan‐cytokeratin (Pan‐CK) and CD10 staining indicated that the majority of these cells were of epithelial origin. NC, negative control. * *p* < 0.05, ^§^ *p* < 0.0001.

To determine whether ectopic endometrial lesions, in addition to the uterine endometrium, contribute to the pool of CECs, we dynamically monitored tdTomato^+^ CECs derived from ectopic lesions in the dual‐color pseudo‐menstrual mouse model (Figure 4A). IVFC revealed a marked increase in tdTomato^+^ CECs during the menstrual phase, reaching 6.38 cells/20 min (*p* < 0.05), closely mirroring the temporal release pattern observed for uterine‐derived EGFP^+^ CECs (Figure 4B).

To further investigate the mechanism underlying this release, two‐photon intravital microscopy was used to visualize cyclical changes in the microvasculature of ectopic lesions. Lesion vessels showed phase‐dependent remodeling, becoming more prominent during the secretory phase and displaying focal disruption during the menstrual phase. These morphological changes paralleled those observed in the uterine endometrium and support the possibility that vascular breakdown in ectopic lesions may represent a second, independent route for CEC entry into the bloodstream (Figure 4C).

tdTomato^+^ and EGFP^+^ CECs showed distinct systemic distribution patterns. IVIS fluorescence imaging detected tdTomato signals in the lungs, but their intensity was markedly lower than the EGFP signals derived from uterine‐origin CECs (Figure 4D). We further performed H&E staining and immunofluorescence on lung sections, which showed that the accumulated CECs in the lung were predominantly Pan‐CK^+^ epithelial cells, supporting an endometrial epithelial‐like phenotype (Figure 4E).

This discrepancy, characterized by comparable circulating abundance but different pulmonary retention, suggests that the fate and biological behavior of CECs may differ according to their origin. Although ectopic lesions, like the uterine endometrium, are capable of releasing cells into the circulation, these tdTomato^+^ CECs appear less likely to survive, adhere, or persist in distant organs such as the lung. Such differences may reflect variation in cellular phenotype, immune recognition, or interactions with the local tissue microenvironment. These findings indicate that both the uterine endometrium and ectopic lesions contribute to the circulating CEC pool during the menstrual phase, but that their systemic fates may diverge substantially.

### 2.5 Uterine‐ and Lesion‐Derived CECs Showed Distinct Postoperative Changes After Removal of the Source Tissues

**FIGURE 5:**
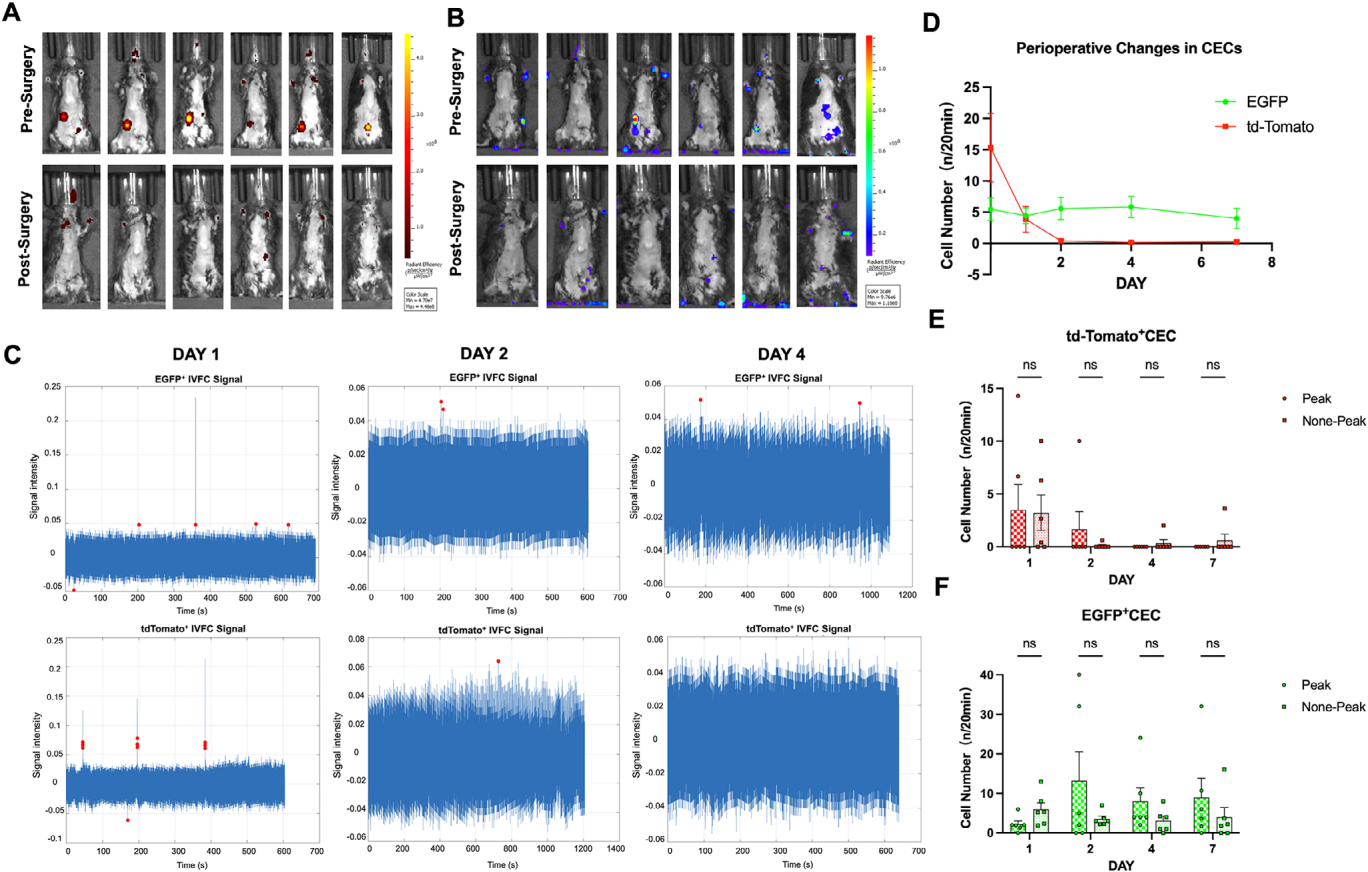
Perioperative changes of CECs after removal of the uterus and ectopic lesions. (A, B) Representative IVIS images acquired before and immediately after surgical removal of the uterus and ectopic lesions in dual‐color pseudo‐menstrual mice. (A) tdTomato and (B) EGFP signals both showed marked reduction in the pelvic region after surgery. For each fluorescence channel, images acquired before and after surgery were displayed using the same pseudocolor scale, indicating successful removal of the major source tissues. (C) Representative IVFC traces showing circulating EGFP^+^ and tdTomato^+^ events at the indicated postoperative time points. (D) Longitudinal changes in circulating EGFP^+^ and tdTomato^+^ events before and after surgery, measured by IVFC. Day 0 indicates the preoperative baseline, whereas days 1, 2, 4, and 7 indicate postoperative follow‐up time points. A significant effect of time and a significant time × fluorophore interaction were observed, whereas the main effect of fluorophore was not significant. tdTomato^+^ events were significantly reduced from postoperative day 1 onward (day 1: *p* = 0.0016; days 2, 4, and 7: all *p* < 0.0001), whereas EGFP^+^ events remained relatively stable. (E, F) Comparison of circulating EGFP^+^ and tdTomato^+^ CEC events between peak and non‐peak periods after surgery. No significant differences were observed between peak and non‐peak periods at the indicated postoperative time points. Data are shown as scatter plots (mean ± SEM, *n* = 6). Statistical analysis was performed using two‐way repeated‐measures ANOVA in (D‐F), followed by Šídák's multiple‐comparison correction. ns, not significant (*p* > 0.05).

To further examine the contribution of the uterus and ectopic lesions to CEC dynamics, six successfully established dual‐color pseudo‐menstrual mice were first evaluated during the menstrual phase to obtain baseline measurements by IVFC and IVIS. The uterus and endometriotic lesions were then surgically removed to eliminate potential source tissues and reservoirs, followed immediately by IVIS imaging. Under identical imaging settings and the same pseudocolor scale, fluorescence signals within anatomically relevant regions, particularly the abdominal region, were markedly reduced immediately after surgery, indicating successful removal of the major fluorescent source tissues (Figure 5A,B). Longitudinal IVFC follow‐up was subsequently performed on postoperative days 1, 2, 4, and 7, revealing distinct postoperative patterns of EGFP^+^ and tdTomato^+^ CECs in the bloodstream.

Overall, tdTomato^+^ CECs in peripheral blood declined markedly after surgery, whereas EGFP^+^ CECs remained relatively stable. Statistical analysis showed that the postoperative trajectories differed significantly between CECs of the two origins. Post hoc comparisons further showed that EGFP^+^ CEC counts did not differ significantly from preoperative levels at any postoperative time point, whereas tdTomato^+^ CEC counts were already significantly lower on postoperative day 1 than before surgery (*p* = 0.0016) and remained low on days 2, 4, and 7 (all *p* < 0.0001) (Figure 5C,D).

These findings suggest that CECs from the two origins differ in their dependence on source tissues. Lesion‐derived tdTomato^+^ CECs appear to rely more strongly on continued release from established lesions, whereas uterine‐derived EGFP^+^ CECs may persist transiently in the circulation even after removal of both the uterus and ectopic lesions. Consistently, the immediate decline in pelvic IVIS signals after surgery and the subsequent origin‐dependent changes in IVFC‐detected CECs support the uterus and ectopic lesions as major sources of circulating CECs.

### 2.6 CEC Release Patterns Were Associated With Prolactin Fluctuations

**FIGURE 6:**
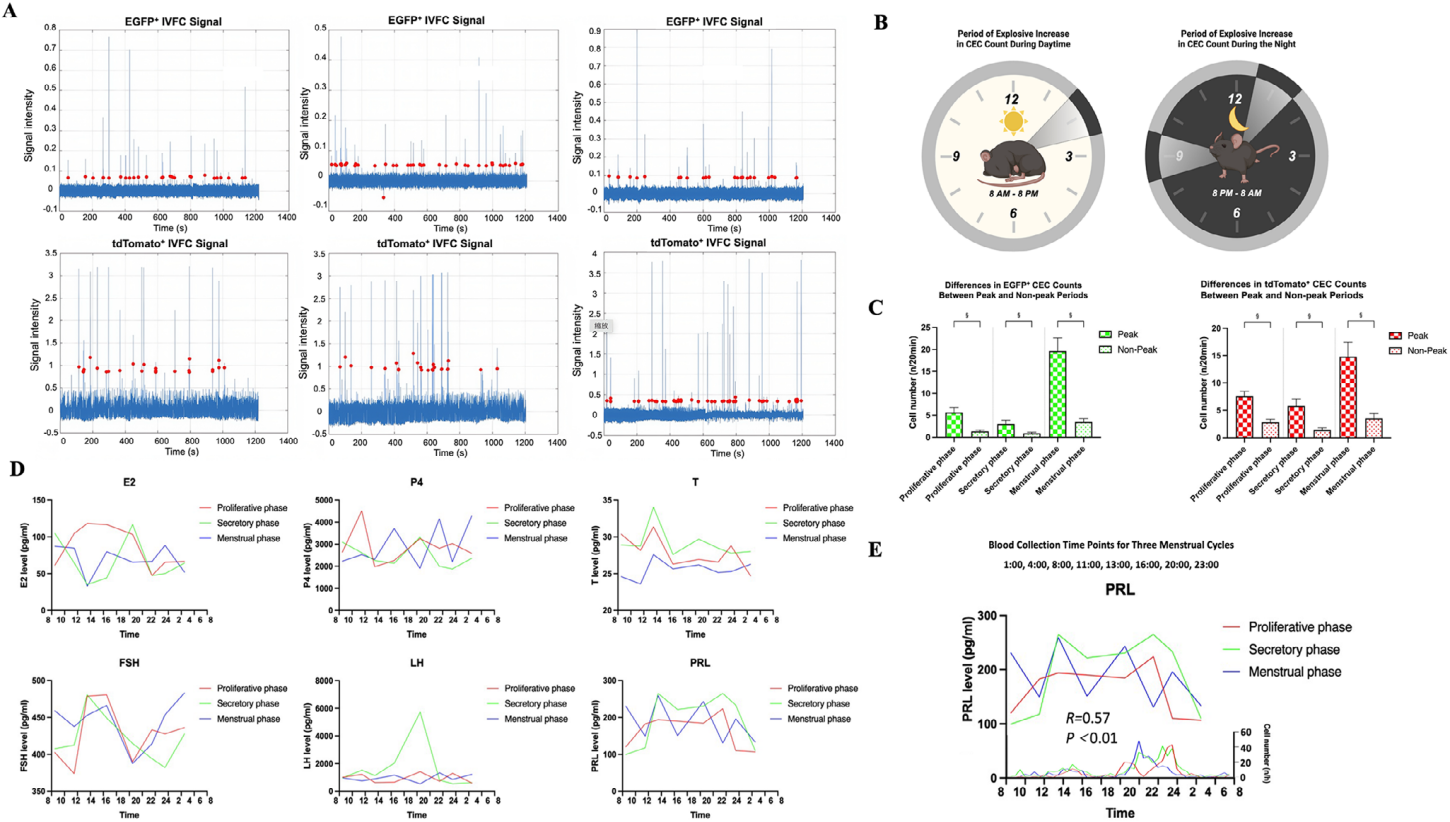
Temporal fluctuations in CEC levels and hormonal correlation. (A) IVFC monitoring revealed burst‐like increases in both EGFP^+^ and tdTomato^+^ CECs at specific time points throughout the day. (B) Graphical summary of the time windows during which CEC levels showed statistically significant increases. These bursts were reproducibly observed within three defined periods across multiple days: 13:30–14:30, 20:30–21:30, and 00:30–01:30. (C) Quantitative comparison of EGFP^+^ and tdTomato^+^ CEC counts between burst and non‐burst periods showed significantly higher CEC levels during burst periods. Data are presented as mean ± SEM (*n* = 6 mice/phase; mixed‐effects model with post hoc multiple‐comparison testing). (D) Hormone fluctuation curves for estradiol (E_2_), progesterone (P_4_), testosterone (T), luteinizing hormone (LH), follicle‐stimulating hormone (FSH), and prolactin (PRL) across the pseudo‐menstrual cycle (mean ± SEM; *n* = 3 mice/time point). (E) Correlation analysis showed that prolactin exhibited the strongest association with CEC fluctuations (R = 0.57, *p* < 0.01), suggesting a potential role in regulating CEC release (Pearson's correlation; *n* = 3 mice/time point). * *p* < 0.05, ^§^ *p* < 0.0001.

To investigate the temporal regulation of CEC release, we performed prolonged IVFC monitoring across a 24 h cycle in the dual‐color pseudo‐menstrual model. Unexpectedly, both uterine‐derived EGFP^+^ CECs and lesion‐derived tdTomato^+^ CECs exhibited concordant burst‐like increases within three recurrent time windows: 13:30–14:30, 20:30–21:30, and 00:30–01:30. Signals detected during these windows were significantly higher than baseline levels during non‐peak periods (*p* < 0.01), suggesting that CEC release may follow a circadian‐like temporal pattern (Figure 6A,B). In contrast, the fibroblast control group showed no comparable time‐clustered pattern, displaying only extremely low‐frequency, scattered, and nonreproducible positive events (Figure S4B).

Quantitative analysis showed that mean CEC counts were significantly higher during peak windows than during non‐peak periods in both uterine‐derived EGFP^+^ and lesion‐derived tdTomato^+^ CECs across the proliferative, secretory, and menstrual phases. During the menstrual phase, EGFP^+^ CEC counts increased from 3.44 cells/20 min during non‐peak periods to 19.63 cells/20 min during peak windows, while tdTomato^+^ CEC counts increased from 3.50 to 14.81 cells/20 min, respectively. The similar temporal pattern observed in the two CEC populations suggests that burst‐like release is a shared feature of CEC dynamics across distinct anatomical origins (Figure 6C).

We next evaluated whether CEC fluctuations differed across cycle stages. IVFC monitoring was aligned with a more refined phase classification, including the early, mid, and late proliferative phases, the early, mid, and late secretory phases, and the menstrual phase (days 1–3). CEC counts peaked during the menstrual phase, particularly on days 1 and 2. In addition, a smaller but consistent increase was also observed during the late proliferative phase (Figure S5). These findings suggest that CEC dissemination may begin before menstrual shedding and become most active during endometrial breakdown. Notably, after removal of the uterus and ectopic lesions, the previously observed differences between peak and non‐peak periods were no longer significant for either type of CEC (Figure 5E,F), further indicating that the temporal release pattern depends on the continued presence of the source tissues.

To explore the hormonal basis of this pattern, we simultaneously measured six major reproduction‐related hormones over the same 24 h timeline: estradiol (E_2_), progesterone (P_4_), testosterone (T), luteinizing hormone (LH), follicle‐stimulating hormone (FSH), and prolactin (PRL) (Figure 6D). Correlation analysis showed that PRL was the only hormone significantly associated with the CEC release pattern (R = 0.57, *p* < 0.01) (Figure 6E). By contrast, estradiol and progesterone showed only weak correlations, whereas testosterone, LH, and FSH showed no meaningful association.

These findings suggest that PRL may be a candidate hormonal regulator of CEC trafficking and bloodstream entry. Given its known roles in immune regulation, angiogenesis, and tissue remodeling, PRL may act as a temporal gating factor that influences the time window during which CECs gain access to the circulation. The temporal CEC peaks identified here, together with their association with prolactin fluctuations, provide a physiological framework for understanding how systemic factors may regulate CEC dynamics and potentially influence the hematogenous dissemination of ectopic cells.

## 3 Discussion

This study establishes a dynamic, high‐resolution in vivo framework for investigating the temporal behavior of CECs in endometriosis and their potential physiological regulatory mechanisms. Within this framework, the dual‐color pseudo‐menstrual mouse model provides a practical platform for simulating endometrial breakdown during the menstrual phase and for tracing endometrial‐related cells from distinct tissue origins, including uterine‐derived and lesion‐derived populations.

Compared with the limitations of conventional rodents, which do not undergo spontaneous menstruation, this model recapitulates menstrual shedding on the basis of hormonal priming and local endometrial manipulation. Notably, the EGFP‐derived endometrial cell‐enriched suspension injected into the uterine cavity did not merely remain transiently within the lumen, but became integrated to some extent into the recipient endometrium, predominantly within the epithelial compartment; the EGFP^+^ epithelial signals subsequently observed in lung tissue also suggest that this phenomenon warrants further attention. More importantly, these EGFP^+^ cells were shed synchronously with the recipient endometrium during the menstrual phase, indicating that they had entered a tissue environment responsive to progesterone withdrawal and local remodeling rather than being passively expelled as free‐floating cells. These features enhance the biological relevance of this model for studying the source and dynamic behavior of CEC release.

When combined with IVFC, this model showed that CECs do not enter the circulation continuously or uniformly, but instead display a clear daily burst‐like release pattern, with reproducible peaks occurring within three distinct time windows. Importantly, this rhythmic increase was observed in both uterine‐derived and lesion‐derived CECs, suggesting the presence of a shared systemic regulatory mechanism rather than a phenomenon restricted to a single tissue source. This temporal fluctuation resembles the circadian‐like dynamics reported for other rare circulating cell populations, including circulating tumor cells, and may have important implications for optimizing sampling strategies and for understanding disease behavior at the systemic level [20].

In addition, after removal of the uterus and ectopic lesions, the two major sources of CECs, CECs from different origins, showed distinct postoperative patterns. The decline in uterine‐derived CECs was not pronounced, which is consistent with our observation that EGFP signals remained detectable in distant organs, whereas tdTomato signals were minimal or nearly absent. This finding suggests that, in addition to the uterus and local lesions, other sites of EGFP^+^ cell retention or storage may persist in vivo and continue to release related signals into the circulation. These results indicate that the in vivo sources of CECs may be more complex than initially anticipated, and that persistence and subsequent re‐release of some uterine‐derived cells in distant tissues may contribute to continued dissemination. From a clinical perspective, this observation suggests that persistent activity of residual cells or microscopic lesions after surgery may warrant further attention. Current guidelines also indicate that postoperative hormonal treatment may be considered for patients who are not seeking immediate pregnancy, in order to reduce the risk of symptom recurrence or disease recurrence [24].

Among the reproductive hormones analyzed, PRL emerged as the most likely candidate regulator of rhythmic CEC release, showing a significant association with CEC peak patterns over time. Beyond its classical lactogenic role, prolactin has increasingly been implicated in tissue remodeling, angiogenesis, and immune regulation, all of which are central to cyclical endometrial regeneration and breakdown. Our findings suggest that prolactin may act as a gating factor that regulates the shedding of endometrial cells from both eutopic and ectopic tissues and their subsequent entry into the vasculature. Targeting the prolactin axis may therefore represent a potential strategy to reduce CEC release and, in turn, limit disease dissemination [25, 26].

The integration of IVFC with the dual‐color model also represents an important methodological advance for tracking rare cell populations in vivo with high temporal resolution. Unlike conventional animal models or routine peripheral blood sampling approaches, this system enables real‐time, longitudinal observation of CECs under minimally invasive conditions, while simultaneously capturing their origin and dynamic changes across the entire cycle [27]. In addition, by visualizing CEC accumulation in distant organs such as the lung and liver, this model allows early steps of hematogenous dissemination to be examined before overt lesion formation, thereby providing a framework for future studies on the determinants of CEC survival, immune evasion, and implantation capacity.

This study has several limitations. First, the primary evidence is derived from an animal model. Although we have identified CECs in human samples, current technical limitations still make it difficult to achieve real‐time dynamic monitoring of CECs in clinical settings, as well as their stable isolation, culture, and systematic characterization. For this reason, we established the dual‐color pseudo‐menstrual mouse model to better understand the role of CECs in endometriosis from a dynamic perspective. Second, the present study examined CEC changes within only a single pseudo‐menstrual cycle, which provides a relatively limited temporal window and is insufficient to fully capture their long‐term fluctuation patterns. In addition, although two‐photon intravital microscopy suggested that CEC entry into the bloodstream may be associated with vascular remodeling and focal barrier disruption during the menstrual phase, a definitive vascular rupture event was not directly recorded because of limitations in imaging resolution and technical constraints. Further work will therefore be needed to refine monitoring and imaging approaches, extend the observation period for tracking CEC dynamics in the dual‐color pseudo‐menstrual model, and improve methods for CEC isolation, culture, and characterization in order to better define their biological properties. Whether this model can be applied to studies of pharmacological intervention also remains to be determined, and prolactin‐related mechanisms require further investigation.

In summary, this study reveals a reproducible, time‐dependent pattern of CEC release and suggests a potential regulatory association with prolactin. It also establishes a robust in vivo model for tracking the dissemination of endometrial cells and defining their tissue origin. Together, these findings provide both a conceptual framework and a technical platform for optimizing diagnostic sampling windows, exploring hormone‐ or rhythm‐based intervention strategies, and advancing our understanding of endometriosis as a systemic disease.

## 4 Materials and Methods

### 4.1 Establishment of the Dual‐Color Pseudo‐Menstrual Mouse Model

To investigate the dynamic behavior of CECs and their relationship with hormonal cyclicity, we established an innovative dual‐color pseudo‐menstrual mouse model using C57BL/6 female mice (Vital River, China) as recipients. This model incorporated EGFP^+^ and tdTomato^+^ endometrial components to trace CEC dissemination, thereby enabling real‐time observation of endometrial shedding and subsequent entry of endometrial cells into the bloodstream.

#### 4.1.1 Ovariectomy and Hormonal Stabilization

On day −14, bilateral ovariectomy was performed in 8–10‐week‐old female C57BL/6 mice to stabilize endogenous hormone levels. After recovery, recipient mice were prepared for endometrial transplantation on day −5. The cervix was reversibly ligated to prevent endometrial cell fragments from being discharged through the vagina, and both oviducts were ligated to prevent retrograde passage of menstrual blood or cell fragments into the pelvic cavity. After decidualization was induced by intrauterine peanut oil injection on day 9, the cervical and oviduct ligatures were removed to restore normal anatomy. This procedure was designed to ensure that the observed bloodstream entry of CECs mainly reflected physiological processes rather than artificial retention caused by luminal obstruction.

#### 4.1.2 Endometrial Tissue Transplantation

Transplants were obtained from two types of donor mice.

**Donor 1**: Endometrial tissue derived from EGFP^+^ transgenic mice (Vitalstar, China) was digested with Collagenase IV (C8160, Solarbio, China) and filtered to obtain a cell suspension. Approximately 2 × 10^5^ cells from this suspension were injected into the uterine cavity of recipient mice to simulate menstrual shedding of the eutopic endometrium.

**Donor 2**: Endometrial tissue fragments (approximately 3 mm in diameter) derived from tdTomato^+^ transgenic mice (Vitalstar, China) were sutured onto the abdominal wall of recipient mice to generate ectopic endometrial lesions and model endometriosis.

#### 4.1.3 Hormonal Induction of Pseudo‐Menstrual Changes

A pseudo‐menstrual cycle was established by subcutaneous administration of estradiol (E808987, Macklin, China) and progesterone (H665445, Macklin, China). Mice received 100 ng estradiol daily on days 1–3. On day 7, 50 ng progesterone and 5 ng estradiol were administered, and a sustained‐release progesterone pump (RWD 1001 W, RWD, China) was implanted. On day 9, mice received 5 ng estradiol and intrauterine peanut oil injection to induce decidualization. On day 11, the progesterone pump was removed to induce progesterone withdrawal–associated endometrial shedding. Based on pilot observations and uterine lavage smear data showing residual shedding at 48 h after withdrawal, days 1–3 after progesterone withdrawal were operationally defined as the menstrual phase in this study.

#### 4.1.4 Vaginal Blood Smears

To confirm successful induction of the menstrual phase in the pseudo‐menstrual model, vaginal blood smears were collected 24 h after progesterone pump removal. Wright–Giemsa staining was performed to examine red blood cells and shed endometrial cells, thereby verifying the presence of a menstrual‐like shedding process.

#### 4.1.5 Uterine Lavage Smears

To further determine whether EGFP^+^ endometrial cells participated in menstrual shedding in a manner similar to endogenous endometrial cells, uterine lavage fluid was collected at 12, 24, and 48 h after hormone pump removal in successfully established dual‐color pseudo‐menstrual mice. Mice without pump removal served as controls, and uterine lavage fluid was collected at the corresponding time point (24 h). The recovered cell suspension was collected in PBS containing 1% FBS (Gibco, USA) and 2 mM EDTA (E1170, Solarbio, China), concentrated by centrifugation, mounted onto positively charged slides, and fixed. Immunofluorescence staining was subsequently performed, and shed cells were phenotypically characterized using anti‐EGFP (GB15603, Servicebio, China) together with the epithelial marker Pan‐cytokeratin (Pan‐CK; 26411‐1‐AP, Proteintech, China) and the stromal marker CD10 (18008‐1‐AP, Proteintech, China).

### 4.2 hEM15A Cells and Fluorescent Labeling

hEM15A is an immortalized stromal cell line derived from eutopic endometrial tissue from a patient with endometriosis [28]. hEM15A cells were cultured in DMEM/F12 medium (Gibco, USA) supplemented with 15% fetal bovine serum (FBS; Gibco, USA) and 1% penicillin/streptomycin. For in vivo tracking, hEM15A cells were labeled with DiD to stain the cell membrane. After labeling, cells were counted using a hemocytometer and adjusted to the desired concentrations to ensure accurate cell numbers for injection. According to the experimental design, the cell suspension was divided into five groups containing 10, 10^2^, 10^3^, 10^4^, and 10^5^ cells, respectively. Cells from each group were injected into mice via the tail vein, with each mouse receiving the corresponding number of cells.

### 4.3 In Vivo Monitoring of CECs

#### 4.3.1 Two‐Photon Intravital Microscopy

Two‐photon intravital microscopy was performed using a TCS‐SP8 DIVE microscope (Leica, Germany). Mice were anesthetized with isoflurane and maintained at a constant temperature during imaging. A midline abdominal incision was made to expose the uterus or abdominal wall endometriotic lesions, and the region of interest was stabilized on the imaging platform for real‐time observation. To visualize vascular structures, PE anti‐mouse CD31 Antibody (102508, BioLegend, USA) was administered via the tail vein before imaging. EGFP^+^ and tdTomato^+^ fluorescence signals were collected to track uterine‐derived and lesion‐derived cells in relation to vascular structures and their dissemination in vivo. All images were processed and analyzed using the accompanying software.

#### 4.3.2 In Vivo Flow Cytometry (IVFC)

An IVFC system was established using the central ear vein of mice to dynamically monitor CECs in the peripheral circulation. This noninvasive approach enabled real‐time recording of fluorescently labeled CEC events (EGFP^+^ and/or tdTomato^+^). Monitoring was performed repeatedly over a 14‐day period at predefined time points, covering three major phases of the cycle, in order to systematically assess fluctuations in CEC abundance across different cycle stages (proliferative, secretory, and menstrual phases) as well as at different times of day. During each recording session, mice were anesthetized with isoflurane, placed on a temperature‐controlled platform, and gently immobilized at the ear to expose the central ear vein and reduce motion artifacts. Each session lasted approximately 10–20 min, with general condition and respiration monitored until recovery.

Because the short‐axis width of the IVFC detection spot is approximately 10 µm and CEC diameter is heterogeneous, ranging from small subpopulations of <5 µm to larger cells of 8–30 µm [14, 15], the effective transit width of a single cell was conservatively approximated as 13–15 µm based on the smaller‐diameter subpopulation in order to estimate the shortest residence time. According to previously reported blood flow parameters in the central ear vein of mice, the local blood flow velocity is approximately 0.2–0.5 cm/s [29, 30], corresponding to a transit time of approximately 2.6–7.5 ms for a single CEC crossing the detection spot. Under an IVFC sampling frequency of 10 kHz, each cell traversing the detection spot would therefore correspond to approximately 26–75 sampling points. Under appropriate threshold and trigger settings, complete failure to sample a passing cell was considered unlikely. Because the depth of field of the objective is limited, cells passing outside the focal plane may generate weaker or incomplete fluorescent signals, thereby limiting IVFC detection accuracy. In practice, this effect was minimized by flattening the ear and fine‐adjusting the focal plane.

Fluorescence‐assisted acquisition was performed using dual 488 nm/561 nm channels, with laser power at the sample plane set to 6 and 18 mW, respectively. PMT voltages were set to 600 and 400 mV, respectively.

For signal processing and event identification, dynamic thresholds were automatically defined using the Pauta method. Only fluorescence peaks with intensities above threshold were considered potential CEC signals, whereas background noise below threshold was excluded. All candidate events then underwent manual secondary inspection to remove false‐positive signals with abnormal width or incomplete waveform despite meeting intensity criteria. By combining automated counting with manual verification, CECs passing through the detection region were measured with improved efficiency and accuracy.

#### 4.3.3 In Vivo Fluorescence Imaging (IVIS)

In addition to IVFC, an IVIS Spectrum system (PerkinElmer, USA) was used to visualize the distribution of fluorescent signals in the lungs, liver, and other tissues of live mice. Mice were anesthetized with isoflurane and subjected to whole‐body fluorescence imaging to assess the extent of CEC dissemination in vivo, with particular attention to the lungs, liver, and abdominal wall lesions.

### 4.4 Hormone Measurements

Within each pseudo‐menstrual cycle, blood samples were collected at eight specific time points (1:00, 4:00, 8:00, 11:00, 13:00, 16:00, 20:00, and 23:00) for hormone measurement. Enzyme‐linked immunosorbent assay (ELISA) kits were used to quantify estradiol (E2; MM‐0566M2, Meimian, China), progesterone (P4; MM‐45704M2, Meimian, China), luteinizing hormone (LH; MM‐44039M2, Meimian, China), follicle‐stimulating hormone (FSH; MM‐45654M2, Meimian, China), testosterone (T; MM‐0569M2, Meimian, China), and prolactin (PRL; MM‐45170M2, Meimian, China). Hormone fluctuation curves were subsequently compared with CEC dynamic profiles to identify potential associations between hormone levels and CEC behavior.

### 4.5 Histological Analysis

Histological evaluation was performed on the lungs, abdominal wall lesions, and uterine tissues. After collection, part of the tissues was fixed in 4% paraformaldehyde and embedded in paraffin, whereas the remaining tissues were embedded in OCT compound for frozen section preparation, in order to assess the tissue distribution of CECs and the formation of ectopic lesions. Hematoxylin and eosin (H&E) staining was used to examine overall tissue morphology. Multiplex immunofluorescence staining based on tyramide signal amplification (TSA) was further performed to detect EGFP^+^ and tdTomato^+^ cells in tissues. To identify endometrial‐derived cells, Pan‐CK and CD10 were used in combination with fluorescence signals to determine the identity of endometrial cells within tissues.

### 4.6 Animal Husbandry and Ethics

All animal experiments in this study were reviewed and approved by the Institutional Animal Care and Use Committee (IACUC) of Peking University People's Hospital (approval no. 2024PHE123) and were conducted in accordance with institutional guidelines for the care and use of laboratory animals and relevant ethical regulations for humane treatment. Mice were housed under controlled environmental conditions with a 12 h light/dark cycle and ad libitum access to food and water. Temperature and humidity were continuously monitored and regulated to ensure animal welfare.

### 4.7 Image Quantification

Image quantification of uterine lavage smears was performed using each mouse as the statistical unit. At the same magnification, 15 nonoverlapping fields were randomly selected from each smear for imaging and counting, and the number of target cells was recorded. The total value across the 15 fields was taken as the final quantitative result for each mouse and used for subsequent statistical analysis.

### 4.8 Statistical Analysis

For uterine lavage smear quantification, each mouse was treated as the statistical unit for intergroup comparisons. Differences among groups were analyzed using one‐way ANOVA or two‐way ANOVA, followed by appropriate multiple‐comparison correction. Statistical analyses of CEC counts and hormone levels were performed to evaluate differences across menstrual‐cycle phases and across different time points within a 24 h period. For repeated measurements obtained from the same animal across different cycle phases and multiple time points, two‐way repeated‐measures ANOVA or mixed‐effects models were used to assess the effects of cycle phase and time of day on CEC counts, followed by multiple‐comparison correction. Correlations between hormone levels and CEC counts were evaluated using Pearson correlation analysis. All statistical analyses were performed using GraphPad Prism 10, and *p* < 0.05 was considered statistically significant.

## Author Contributions

Conceptualization: S.W., C.Y.H., H.Y.C., X.H.C., X.B.W., and H.L.Z.; Methodology: S.W., C.Y.H., X.B.W., H.Y.C., B.Y.L., X.Y., Y.M.L., S.H.D. and R.Z.; Visualization: S.W. and C.Y.H.; Funding acquisition: X.H.C., X.B.W., H.L.Z., and Y.L.; Project administration: S.W., H.Y.C., X.H.C., and H.P.L.; Supervision: C.Z. and H.Y.H.; Writing – original draft: S.W., C.Y.H., X.B.W., and X.H.C.; Writing – review & editing: S.W., C.Y.H., H.Y.C., X.H.C., X.B.W., B.Y.L., and H.L.Z.

## Funding

This work was supported by grants from the National Natural Science Foundation of China (No. 82471683 and No. 81971360), the National Key Research and Development Program of China (No. 2022YFC2704001 and No. 2021YFF0502900), and the Special Fund for Research on National Major Research Instruments of China (No. 62027824).

## Conflicts of Interest

The authors declare no conflicts of interest.

## Supporting information




**Supporting File**: advs76327‐sup‐0001‐SuppMat.docx.

**Supporting movie 1**: advs76327‐sup‐0002‐MovieS1.avi.

## Data Availability

The data that supports the findings of this study are available in the supplementary material of this article.

## References

[1] S. As‐Sanie , S. C. Mackenzie , L. Morrison , et al., “Endometriosis,” JAMA 334, no. 1 (2025): 64, 10.1001/jama.2025.2975. 40323608 PMC13498397

[2] T. Hirono , Y. Feng , W. Wang , and H. Yu , “Spontaneous Recurrent Menstrual Pneumothorax: a Case Report,” Annals of Medicine & Surgery 86, no. 2 (2024): 1096–1100, 10.1097/MS9.0000000000001592. 38333324 PMC10849425

[3] W. Yin , X. Li , P. Liu , et al., “Digestive System Deep Infiltrating Endometriosis: What Do We Know,” Journal of Cellular and Molecular Medicine 27, no. 23 (2023): 3649–3661, 10.1111/jcmm.17921. 37632165 PMC10718155

[4] A. Tsuei , F. Nezhat , N. Amirlatifi , Z. Najmi , A. Nezhat , and C. Nezhat , “Comprehensive Management of Bowel Endometriosis: Surgical Techniques, Outcomes, and Best Practices,” Journal of Clinical Medicine 14, no. 3 (2025): 977, 10.3390/jcm14030977. 39941647 PMC11818743

[5] M. Meggyesy , M. Friese , J. Gottschalk , and U. Kehler , “Case Report of Cerebellar Endometriosis,” Journal of Neurological Surgery Part A: Central European Neurosurgery 81, no. 04 (2020): 372–376, 10.1055/s-0040-1701622. 32221961

[6] J. W. K. Lee , S. S. Tang , J. L. Ong , H. B. Oh , and B. Lieske , “Perianal Endometriosis: an Unusual Cause of Perianal Pain,” Gynecology and Obstetrics Clinical Medicine 1, no. 4 (2021): 225–227, 10.1016/j.gocm.2021.10.002.

[7] K. T. Zondervan , C. M. Becker , and S. A. Missmer , “Endometriosis,” New England Journal of Medicine 382, no. 13 (2020): 1244–1256, 10.1056/NEJMra1810764. 32212520

[8] J. Y. Shim , “Dysmenorrhea and Endometriosis in Adolescents,” Obstetrics and Gynecology Clinics of North America 51, no. 4 (2024): 651–661, 10.1016/j.ogc.2024.08.003. 39510736

[9] P. R. Koninckx , R. Fernandes , A. Ussia , et al., “Pathogenesis Based Diagnosis and Treatment of Endometriosis,” Frontiers in Endocrinology 12 (2021): 745548, 10.3389/fendo.2021.745548. 34899597 PMC8656967

[10] S. Rajesh , A. Mehmeti , T. Smith‐Walker , and B. Kendall , “Diagnosis and Management of Endometriosis: Summary of Updated NICE Guidance,” Bmj 388 (2025): q2782, 10.1136/bmj.q2782. 39890103

[11] G. Andreani , M. C. V. Lauar , A. K. Medeiros , and C. C. Barbisan , “Diagnosis and Laparoscopic Management of Intrahepatic Endometrioma,” Journal of Minimally Invasive Gynecology 32, no. 11 (2025): 945–947, 10.1016/j.jmig.2025.02.007. 39956449

[12] M. P. Andres , F. V. L. Arcoverde , C. C. C. Souza , L. F. C. Fernandes , M. S. Abrão , and R. M. Kho , “Extrapelvic Endometriosis: a Systematic Review,” Journal of Minimally Invasive Gynecology 27, no. 2 (2020): 373–389, 10.1016/j.jmig.2019.10.004. 31618674

[13] J. Jarrell , “The Significance and Evolution of Menstruation,” Best Practice & Research Clinical Obstetrics & Gynaecology 50 (2018): 18–26, 10.1016/j.bpobgyn.2018.01.007. 29530426

[14] S. Wang , H. Cheng , H. Zhu , X. Yu , X. Ye , and X. Chang , “Precise Capture of Circulating Endometrial Cells in Endometriosis,” Chinese Medical Journal 137, no. 14 (2024): 1715–1723, 10.1097/cm9.0000000000002910. 38679794 PMC11268826

[15] Y. Chen , H. L. Zhu , Z. W. Tang , et al., “Evaluation of Circulating Endometrial Cells as a Biomarker for Endometriosis,” Chinese Medical Journal 130, no. 19 (2017): 2339–2345, 10.4103/0366-6999.215325. 28937041 PMC5634086

[16] O. D. Slayden , F. Luo , and D. V. M. L. Martin , “A Protocol for Creating Endometriosis in Rhesus Macaques ( Macaca mulatta ),” Journal of Medical Primatology 52, no. 6 (2023): 405–413, 10.1111/jmp.12681. 37849073 PMC10843508

[17] K. A. Burns , A. M. Pearson , J. L. Slack , et al., “Endometriosis in the Mouse: Challenges and Progress toward a ‘Best Fit’ Murine Model,” Frontiers in Physiology 12 (2021): 806574, 10.3389/fphys.2021.806574. 35095566 PMC8794744

[18] L. H. Kennedy , M. H. Nowland , and J. A. Nemzek‐Hamlin , “Surgical Treatment of Spontaneous Endometriosis in Rhesus Macaques (Macaca mulatta): 11 Cases (2007–2011),” Journal of the American Veterinary Medical Association 254, no. 12 (2019): 1454–1458, 10.2460/javma.254.12.1454. 31149880

[19] R. C. Wilson , J. M. Link , Y. Z. Lee , J. D. Oldan , S. L. Young , and O. D. Slayden , “Uterine Uptake of Estrogen and Progestogen‐Based Radiotracers in Rhesus Macaques with Endometriosis,” Molecular Imaging and Biology 26, no. 2 (2024): 334–343, 10.1007/s11307-023-01892-9. 38133866 PMC11034810

[20] X. Zhu , Y. Suo , Y. Fu , et al., “In Vivo Flow Cytometry Reveals a Circadian Rhythm of Circulating Tumor Cells,” Light: Science & Applications 10, no. 1 (2021): 110, 10.1038/s41377-021-00542-5. PMC8160330 34045431

[21] F. Zhang , X. Lu , X. Zhu , Z. Yu , W. Xia , and X. Wei , “Real‐Time Monitoring of Small Extracellular Vesicles (sEVs) by in Vivo Flow Cytometry,” Journal of Extracellular Vesicles 13, no. 10 (2024): 70003, 10.1002/jev2.70003. PMC11497658 39441010

[22] M. Lee , S. Kannan , G. Muniraj , et al., “Two‐Photon Fluorescence Microscopy and Applications in Angiogenesis and Related Molecular Events,” Tissue Engineering Part B: Reviews 28, no. 4 (2022): 926–937, 10.1089/ten.TEB.2021.0140. 34541887

[23] M. Brasted , C. A. White , T. G. Kennedy , and L. A. Salamonsen , “Mimicking the Events of Menstruation in the Murine Uterus,” Biology of Reproduction 69, no. 4 (2003): 1273–1280, 10.1095/biolreprod.103.016550. 12801986

[24] C. M. Becker , A. Bokor , O. Heikinheimo , et al., “ESHRE Guideline: Endometriosis,” Human Reproduction Open 2022, no. 2 (2022): hoac009, 10.1093/hropen/hoac009. 35350465 PMC8951218

[25] G. J. Lee , F. Porreca , and E. Navratilova , “Prolactin and Pain of Endometriosis,” Pharmacology & Therapeutics 247 (2023): 108435, 10.1016/j.pharmthera.2023.108435. 37169264 PMC12181984

[26] C. Otto , H.‐F. Ulbrich , and C. Freiberg , “The Effects of Prolactin Receptor Blockade in a Murine Endometriosis Interna Model,” Pharmacology Research & Perspectives 10, no. 1 (2022): 00916, 10.1002/prp2.916. PMC8929327 35084123

[27] K. Wang , X. Wang , Q. Pan , and B. Zhao , “Liquid Biopsy Techniques and Pancreatic Cancer: Diagnosis, Monitoring, and Evaluation,” Molecular Cancer 22, no. 1 (2023): 167, 10.1186/s12943-023-01870-3. 37803304 PMC10557192

[28] Y. Chen , Y. H. Yi , H. J. Han , et al., “Establishment of Human Endometriosis‐derived Immortalized Eutopic Endometrium Stromal and Epithelial Cell Lines,” International Journal of Clinical and Experimental Medicine 9, no. 8 (2016): 16450.

[29] E. I. Galanzha , M. G. Viegas , T. I. Malinsky , et al., “In Vivo Acoustic and Photoacoustic Focusing of Circulating Cells,” Scientific Reports 6 (2016): 21531, 10.1038/srep21531. 26979811 PMC4793240

[30] L. Chao , C. Jiangbo , Z. Yachao , Z. Jingyi , and W. Lidai , “Five‐Wavelength Optical‐Resolution Photoacoustic Microscopy of Blood and Lymphatic Vessels,” Advanced Photonics 3, no. 1 (2021): 016002, 10.1117/1.AP.3.1.016002.

