# Supplementary material for: Real‐Time in Vivo Monitoring of Circulating Endometrial Cells: Uncovering Systemic Dissemination and Temporal Fluctuations in Endometriosis
Source: Adv Sci (Weinh). 2026 Jun 26;13(53):e76327. doi: 10.1002/advs.76327 (PMC13336561; doi:10.1002/advs.76327)
Supplement: Supplementary file 1 [file ADVS-13-e76327-s002.docx]

Supporting Information

**Real-time In Vivo Monitoring of Circulating Endometrial Cells: Uncovering Systemic Dissemination and Temporal Fluctuations in Endometriosis**

*Shang Wang, Chenyu Hou, Buyun Li, Hongyan Cheng, Xue Ye, Honglan Zhu, Yanmin Li, Rui Chen, Sihan Dong, Yi Li, Huiping Liu, Chen Zhang, Rui Zhang, Hongyi Hou, Xunbin Wei***, Xiaohong Chang**

Shang Wang, Chenyu Hou, Buyun Li, Hongyan Cheng, Xue Ye, Honglan Zhu, Yi Li, Huiping Liu, Chen Zhang, Hongyi Hou, Xiaohong Chang

Department of Obstetrics and Gynecology, Peking University People’s Hospital, Beijing, China

Beijing Key Laboratory of Female Oncofertility, Beijing, China

Yanmin Li, Rui Chen, Sihan Dong, Rui Zhang, Xunbin Wei

Key Laboratory of Carcinogenesis and Translational Research (Ministry of Education/Beijing), Peking University Cancer Hospital & Institute, Beijing, China

Biomedical Engineering Center, Institute of Advanced Clinical Medicine, Peking University, Beijing, China

Peking University International Cancer Institute, Beijing, China

Institute of Medical Technology and Cancer Hospital, Peking University, Beijing, China

Beijing Advanced Center of Cellular Homeostasis and Aging-Related Diseases, Beijing, China

* Correspondence should be addressed to: Xiaohong Chang (changxiaohong@pkuph.edu.cn) or Xunbin Wei ([xwei@bjmu.edu.cn](mailto:xwei@bjmu.edu.cn))

**Shang Wang, Chenyu Hou, Buyun Li, and Hongyan Cheng contributed equally to this work.**

**Figure S1. Schematic representation of the CEC hypothesis in endometriosis.**

Schematic illustration of the circulating endometrial cell (CEC) hypothesis in endometriosis. Endometrial cells may enter the bloodstream through disrupted or remodeled endometrial vasculature during the menstrual phase. Once in circulation, CECs may be cleared, transiently retained, or disseminated to distant organs such as the lungs or liver, where they may accumulate and potentially contribute to lesion formation. Established ectopic lesions may also release CECs back into the circulation, thereby contributing to continued dissemination. The CEC hypothesis is proposed as a complementary mechanism that may coexist with retrograde menstruation. ***Created in BioRender. https://BioRender.com/8ioq0r5.***

**Figure S2. DiD labeling of hEM15A cells reveals distinct red fluorescence.**

hEM15A cells were labeled with the DiD membrane dye and examined by confocal microscopy. Clear red fluorescence was observed in nearly all analyzed cells, indicating highly efficient labeling. These results support the use of DiD-labeled hEM15A cells for subsequent IVFC detection.

**Figure S3. Sensitivity of IVFC for detecting low numbers of circulating hEM15A cells in mice.**

DiD-labeled hEM15A cells were intravenously injected into mice at doses of 10, 10², 10³, 10⁴, and 10⁵ cells, together with a control group without cell injection. Fluorescent events in the bloodstream were monitored in real time by in vivo flow cytometry (IVFC) for 30 min. As the number of injected cells decreased, the number of detected events also declined. Detectable signals were still observed at the lowest cell doses, supporting the sensitivity of IVFC for detecting very small numbers of circulating cells in vivo.

**Figure S4. IVFC monitoring of EGFP⁺ fibroblasts shows rare and scattered signals in control mice.**

**(A)** Representative EGFP-channel IVFC traces from fibroblast-injected mice. Fibroblast-injected mice were subjected to the same pseudo-menstrual hormone regimen and monitored during the proliferative phase by IVFC for 20 min per recording. Most recordings remained at background level, with only occasional isolated positive peaks detected (red dots).

**(B)** Numbers of positive and negative recordings during peak and non-peak periods. Positive recordings were defined as monitoring sessions containing at least one detectable EGFP⁺ event. A total of five control mice were analyzed (n = 5 mice), with 15 peak-period and 15 non-peak-period recordings. Positive signals were observed in 3/15 peak-period recordings and 1/15 non-peak-period recordings, indicating that fibroblast-derived events were rare, scattered, and not enriched within specific time windows.

**Figure S5. CEC counts across the cycle.**

Circulating endometrial cell (CEC) counts were analyzed across different cycle phases, including the early, mid, and late proliferative phases, the early, mid, and late secretory phases, and menstrual days 1, 2, and 3. The highest CEC levels were observed during the menstrual phase, particularly on days 1 and 2.
